# Breathe Easier Online: Evaluation of a Randomized Controlled Pilot Trial of an Internet-Based Intervention to Improve Well-being in Children and Adolescents With a Chronic Respiratory Condition

**DOI:** 10.2196/jmir.1997

**Published:** 2012-02-08

**Authors:** Peter A Newcombe, Tamara L Dunn, Leanne M Casey, Jeanie K Sheffield, Helen Petsky, Sophie Anderson-James, Anne B Chang

**Affiliations:** ^1^School of PsychologyUniversity of QueenslandBrisbaneAustralia; ^2^Griffith Health InstituteBehavioural Basis of HealthGriffith UniversityBrisbaneAustralia; ^3^Queensland Children’s Respiratory CentreQueensland Children’s Medical Research InstituteRoyal Children’s HospitalBrisbaneAustralia; ^4^Child Health DivisionMenzies School of Health ResearchDarwinAustralia

**Keywords:** Internet-based intervention, chronic respiratory condition, psychosocial well-being, children and adolescents, randomized controlled trial

## Abstract

**Background:**

Chronic respiratory illnesses are the most common group of childhood chronic health conditions and are overrepresented in socially isolated groups.

**Objective:**

To conduct a randomized controlled pilot trial to evaluate the efficacy of Breathe Easier Online (BEO), an Internet-based problem-solving program with minimal facilitator involvement to improve psychosocial well-being in children and adolescents with a chronic respiratory condition.

**Methods:**

We randomly assigned 42 socially isolated children and adolescents (18 males), aged between 10 and 17 years to either a BEO (final n = 19) or a wait-list control (final n = 20) condition. In total, 3 participants (2 from BEO and 1 from control) did not complete the intervention. Psychosocial well-being was operationalized through self-reported scores on depression symptoms and social problem solving. Secondary outcome measures included self-reported attitudes toward their illness and spirometry results. Paper-and-pencil questionnaires were completed at the hospital when participants attended a briefing session at baseline (time 1) and in their homes after the intervention for the BEO group or a matched 9-week time period for the wait-list group (time 2).

**Results:**

The two groups were comparable at baseline across all demographic measures (all *F* < 1). For the primary outcome measures, there were no significant group differences on depression (*P* = .17) or social problem solving (*P* = .61). However, following the online intervention, those in the BEO group reported significantly lower depression (*P* = .04), less impulsive/careless problem solving (*P* = .01), and an improvement in positive attitude toward their illness (*P* = .04) compared with baseline. The wait-list group did not show these differences. Children in the BEO group and their parents rated the online modules very favorably.

**Conclusions:**

Although there were no significant group differences on primary outcome measures, our pilot data provide tentative support for the feasibility (acceptability and user satisfaction) and initial efficacy of an Internet-based intervention for improving well-being in children and adolescents with a chronic respiratory condition.

**Trial registration:**

Australian New Zealand Clinical Trials Registry number: ACTRN12610000214033; http://www.anzctr.org.au/trial_view.aspx?ID=308074 (Archived by WebCite at http://www.webcitation.org/63BL55mXH)

## Introduction

Chronic respiratory illnesses include asthma and cystic fibrosis, and are the most common group of childhood chronic conditions [1]. Further, these conditions are more widespread in socially isolated and disadvantaged children and adolescents than they are in higher socioeconomic groups, even after adjusting for traditional risk factors [2,3]. Children and adolescents with a chronic respiratory condition often feel different from their healthy peers due to the necessity of a daily treatment regimen; can have trouble maintaining friendships because of school absences [4]; and can experience psychological difficulties such as anxiety, depression, and poor quality of life (for review, see [5]). They also tend to be involved in risky behaviors such as smoking and to make poor lifestyle choices.

Interventions that enhance self-management skills with an emphasis on monitoring and managing health impacts can optimize physical and psychosocial functioning. Meta-analyses [6,7] show that face-to-face psychological interventions in pediatric chronic medical illnesses promote adherence and improve health outcomes. These interventions have large effect sizes irrespective of illness type, severity, and duration [8]. For treatment adherence, behavioral and multicomponent therapies have been shown to be more effective than educational programs alone [9]. The multicomponent approaches included combinations of a wide range of psychological therapies incorporating problem-solving, family behavioral therapy, biofeedback, social skills training, and psychosocial interventions.

Two recent studies [10,11] have investigated the efficacy of problem-solving skills training in improving well-being in children with a chronic respiratory condition (asthma) and from low-income families. For both, the training, delivered either at the individual family level [11] or as group lessons [10], involved face-to-face interactive problem-solving activities including defining problems, finding alternative solutions, and evaluating solutions. There was some support for the efficacy of the intervention with improved child academic performance and self-regulation [10] and health-related well-being [11] noted.

While face-to-face psychological interventions for adolescents with chronic health conditions are available in most hospitals, the limited availability and accessibility of such programs is a substantial barrier to more widespread uptake of routine psychosocial treatment or preventive interventions. The Internet offers a dynamic, interactive medium for providing information, changing attitudes and behavior, and enhancing social support [12]. It can create contexts where young people can feel safe enough to participate in activities and talk about issues important to them with other young people in similar situations. Despite their appeal and potential, Internet-based interventions for those with chronic health conditions are still quite novel. Stinson et al [13] systematically reviewed nine Internet interventions for pediatric conditions including asthma, pain, encopresis, obesity, and traumatic brain injury. They found positive results for Internet self-management interventions across a range of outcomes related to knowledge, behavioral change, and symptom management, but were unable to conclude that Internet interventions were effective in improving self-efficacy, social support, or emotional well-being. They also noted, however, that more rigorous randomized controlled trials were needed to endorse the use of Internet interventions.

In addition to the lack of robust randomized controlled trial data, many of these Internet-based interventions aimed to improve self-management only and did not provide peer social support. They did not address well-being in terms of increasing social support from others with a similar health condition or teaching skills for addressing life problems, but simply focused on problems associated with self-management. Increasing social support was a primary aim of one pilot study involving young people, aged between 13 and 18 years, with cystic fibrosis [14]. This Internet-based intervention consisted of an online support group including forums, email facility, and a graffiti wall. However, there were no chat facilities, so the communication between the participants was asynchronous. The adolescents in the study reported that they enjoyed emailing each other and going to the website, and were significantly more likely than before to respond that they had a friend with cystic fibrosis to whom they could relate following the intervention. Although promising, the findings of this study are limited, as it was not a randomized controlled trial and had no control group.

### Present Study

Based on the finding that multicomponent interventions are more successful than educational interventions alone [9], our study incorporated social support and problem-solving skills to improve psychosocial well-being in children and adolescents with a chronic respiratory condition. The problem-solving paradigm was based on the work of D’Zurilla and Nezu [15], who framed problem solving as a cognitive–behavioral process whereby individuals endeavor to focus their coping efforts on altering the problematic nature of the situation, their reactions to the situation, or both. We developed an Internet intervention, Breathe Easier Online (BEO), specifically to improve social support and problem-solving skills. The intervention consisted of two parts: structured modules that the participants completed on a weekly basis to improve their social problem-solving skills; and an online community that incorporated both synchronous and asynchronous communication opportunities.

The aim of our study was to evaluate the efficacy of an online intervention for socially isolated children and adolescents with a chronic respiratory illness to improve psychosocial well-being. We hypothesized that, in this pilot randomized controlled trial, those receiving the BEO intervention would show improved psychological well-being (as evidenced by lower depression scores) and problem-solving skills following intervention compared with those in a wait-list control group. We also anticipated positive secondary health-related outcomes for the BEO group, including an improved attitude toward their illness and improved treatment adherence.

## Methods

### Participants

We recruited 42 children aged between 10 and 17 years (mean age 13.58, SD 1.92 years) from the respiratory outpatient clinic at the Royal Children’s Hospital, Brisbane, Australia. Children were eligible to participate if they spoke English as their primary language, had a primary diagnosis of a chronic respiratory condition, did not have any cognitive or sensory impairment that would preclude their completion of study measures, and were deemed socially isolated or disadvantaged by hospital staff based on social indicators (single parent who is unemployed or employed part-time, and known psychological and/or financial difficulties from hospital social work records). Exclusion criteria were children who were unable to use a computer, had an underlying psychiatric disorder, or had a recent (<3 months) hospitalization.

Ethical approval was granted by the Behavioral and Social Sciences Ethical Review Committee at the University of Queensland and the Human Ethics Committee of the Royal Children’s Hospital, Brisbane. Informed consent was obtained from both the parent and participant in those aged 12 years or older and from parents alone in children aged under 12 years.

### Protocol

Potential participants and their families were identified through hospital records by a research nurse at the hospital between July 2008 and November 2009, and were invited to participate. Following completion of the consent forms, children were randomly allocated to one of the two conditions: Internet-based intervention (BEO) or a wait-list control. Order of random allocation was predetermined via a computer program and was unknown to and concealed from the research staff. After baseline assessment (time 1) including completion of the package of questionnaires and spirometry, those in the BEO group received a Toshiba notebook computer and a modem to provide broadband Internet access for the duration of the study. Percentage of predicted forced expiratory volume in 1 second was measured with a spirometer that provides an objective measure of severity of obstructive lung diseases. The predicted value is based on Australian norms and depends on the child’s height, age, and gender.

Paper-and-pencil questionnaire packages were completed at the hospital or at participants’ homes at two time points (time 1: baseline/preintervention, and time 2: postintervention) for the BEO condition and at time equivalents to pre- and postintervention (9 weeks later) for the wait-list group. At time 2, the wait-list group was invited to participate in the intervention.

### The Questionnaire Package

This consisted of several measures chosen to evaluate changes in the participants’ psychological well-being (operationalized by a measure of depressive symptomatology), problem solving, attitudes toward their illness, and opinions and satisfaction with the intervention.

#### Center for Epidemiologic Studies Depression Scale for Children

The Center for Epidemiologic Studies Depression Scale for Children (CES-DC) [16] is a 20-item, self-report depression inventory with each item (eg, “During the past week, I felt like I was too tired to do things”) rated on a 4-point Likert scale (0, not at all; 1, a little; 2, some; 3, a lot). Higher scores reflect higher depressive symptoms, with scores greater than 15 reflecting significant depressive symptoms [16]. The CES-DC has good internal consistency (alpha = .89) [16]. Cronbach alpha = .89 for the current sample.

#### Social Problem-Solving Inventory–Revised (Short Form)

The Social Problem-Solving Inventory–Revised (Short Form) (SPSI-R:SF) [17] comprises 25 items proposed to load onto 5 scales: positive problem orientation (eg, “Whenever I have a problem, I believe it can be solved”), negative problem orientation (eg, “I feel nervous and unsure of myself when I have an important decision to make”), rational problem solving (eg, “When I have a problem to solve, one of the first things I do is get as many facts about the problem as possible”), impulsive/careless style (eg, “I am too impulsive when it comes to making decisions”), and avoidance style (eg, “I go out of my way to avoid having to deal with problems in my life”). Items are rated on a 5-point Likert scale (0, not at all true of me; 1, slightly true of me; 2, moderately true of me; 3, very true of me; 4, extremely true of me) in which participants are asked to indicate how they would usually respond to problems. Higher scores on each scale indicate greater intensity on that attribute. The SPSI-R:SF has good internal consistency (alpha = .85 for adolescents) [17]. Cronbach alpha = .74 for the current sample.

#### Child Attitude Toward Illness Scale

The Child Attitude Toward Illness Scale (CATIS) [18] comprises 13 items designed to assess how favorably or unfavorably children feel toward having a chronic health condition (eg, “How often do you feel different from others because of your breathing problem?”) on a 5-point Likert scale (1, never; 2, not often; 3, sometimes; 4, often; 5, very often). Higher scores reflect more positive attitudes toward illness. The CATIS has demonstrated good internal consistency (alpha = .77 to alpha = .89) and test–retest reliability over a 2-week interval (*r* = .77 to *r* = .80) in children and adolescents aged 8 to 17 years with epilepsy and asthma [18,19]. Cronbach alpha = .85 for the current sample.

#### Intervention Satisfaction Scale

This was a purpose-created self-report scale designed to explore the participants’ opinions and satisfaction with the intervention, as well as seeking feedback at time 2. They rated 8 items (eg, “Would you recommend the program to others?”) on a 4-point Likert scale (1, yes, very much so; 2, yes, for the most part; 3, no, not really; 4, no, not at all). A final open-ended item allowed for any general comments or suggestions about the BEO intervention.

### The BEO program

**Figure 1:**
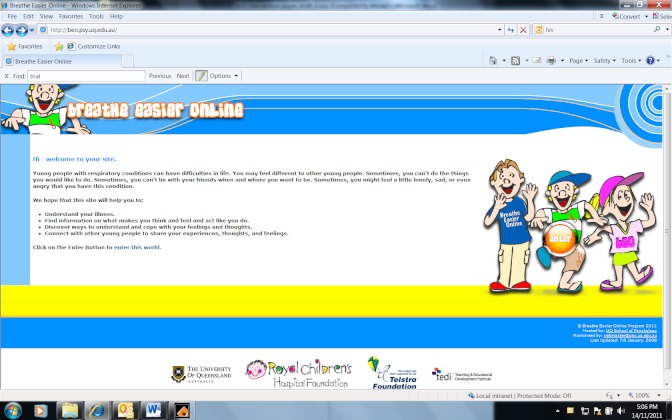
Screenshot of Breathe Easier Online home page.

The interactive website for the participants in the BEO group (see Figure 1) was password protected and consisted of 5 components: My condition, My page, Daily diary, My work, and My talk.

*My condition* is a brief summary of each of the respiratory conditions that provided children with information about their own condition and conditions of the other children they encountered in the BEO program.

In *My page*, participants posted information about themselves including demographics, a photo (if they wished), favorite movie, favorite band, and a brief story about themselves. This page was visible to other BEO participants.

The *Daily diary* section contained a checklist where participants noted the medications they had taken each day. They also recorded how often they conversed with other participants in the program.

The *My work* section of the website contained the 6 modules that formed the focal intervention. The modules were based on D’Zurilla and Nezu’s problem-solving theory [15] and provided interactive online skills training that targeted problems in general in addition to illness-specific problems. The online package followed a PACE principle for solving problems: problem identification, alternative solution generation, consequences of each alternative solution, execute solution and evaluate.

The *My talk* component of the website provided opportunities for BEO participants to communicate with each other. This communication could be either asynchronous (discussion board, email) or synchronous (instant messenger).

The 6 modules progressively and systematically introduced the participants to understanding their condition and treatment, the PACE program, and thinking processes. The intervention was interactive, as participants completed and submitted homework and were able to communicate online and regularly with research staff and other participants.

### Statistical Analysis

Sample size was based on an expected medium effect size of change in psychosocial measures for the intervention group with statistical power set at 90% and alpha =.05. We used SPSS version 17 (IBM Corporation, Somers, NY, USA) to analyze the data. An intention-to-treat approach to analyses was adopted with all participants included in the final analysis. The intention-to-treat analysis (missing values replaced with linear trend) ensured that those participants who completed time 1 measures and the intervention (but did not complete all time 2 measures) were included in the analyses. We used descriptive statistics to describe characteristics of the sample. Linear mixed models were used to assess the effects of the intervention on outcomes using a mixed-model analysis of variance (ANOVA) approach with time (time 1 vs time 2) as a within-subjects factor and group (BEO vs wait-list) as a between-subjects factor. Follow-up analyses were conducted in line with the stated hypotheses. The criterion for statistical significance was set at a 2-tailed value of *P* ≤ .05.

## Results

### Characteristics of Participants

**Figure 2:**
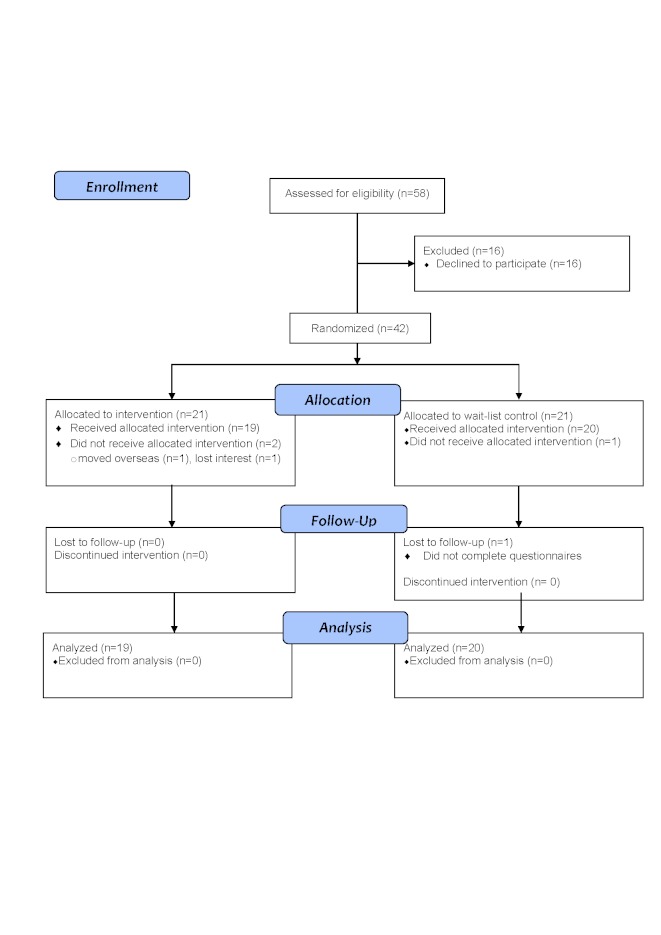
CONSORT flow diagram for the randomized controlled trial of the Breathe Easier Online intervention.

The flow diagram of those we approached and recruited, and who dropped out is depicted in Figure 2. Of the 42 participants who agreed to participate, 39 completed the trial, and 2 dropped out from the BEO intervention condition (final n = 19) and 1 from the wait-list control condition (final n = 20). All participants were white and 19/39 (49%) were male. Of the 39 participants, 12 (31%) had asthma, 22 (56%) had cystic fibrosis, 1 (3%) had tracheomalacia, and 2 (5%) had bronchiectasis. Table 1 presents the data relating to demographic and respiratory conditions of the participants. There were no significant differences between the wait-list control and BEO intervention groups on any of the demographic, respiratory condition, or spirometry results at baseline (time 1; all *F* < 1). Of the 39 participants in the study, 35 were from single-parent families, with the majority (n = 20, 57%) not working. In addition, 13 families were living in rented accommodation and 10 families reported significant financial and/or psychological issues. A total of 12 of the children (31%) had reported poor adherence to treatment.

**Table 1:** Sample characteristics at baseline for both wait-list control and Breathe Easier Online (BEO) intervention conditions

		Wait-list control (n = 20)	BEO intervention (n = 19)	*P* value^a^
**Age (years)**				
	Mean (SD)	13.63 (1.83)	13.41 (1.99)	.71
**Gender**				
	Male/female	10/10	9/10	.87
**Respiratory condition**				
	Cystic fibrosis	9	13	
	Asthma	7	5	
	Other	4	1	.24
**Spirometry measures**				
	FEV_1_^b^, % predicted	74.19 (26.31)	78.17 (25.82)	.63
	FVC^c^, % predicted	85.10 (26.52)	89.37 (26.76)	.61
	FEF_25%–75%_ (L/min)^d^	61.32 (27.72)	62.71 (27.28)	.87

^a^ *P* values for *t* tests of group differences.

^b^ Forced expiratory volume in 1 second.

^c^ Forced vital capacity.

^d^ Forced expiratory flow, midexpiratory phase.

### Effect of BEO Intervention

The descriptive data for the mixed-models ANOVAs are presented in Table 2, showing primary outcomes of depressive symptoms and social problem-solving skills, and secondary outcomes of attitude toward illness and spirometry (as an indicator of treatment adherence). The results for the analyses of depression scores revealed neither significant group (*F*_1,33_ = 2.00, *P* = .17, η^2^ = .06) or time (*F* < 1, *P* = .35, η^2^ = .03) main effects, nor a significant interaction (*F*_1,33_ = 1.76, *P* = .19, η^2^ = .05). Similar results were found for full scale scores on social problem solving with neither significant main effects for group (*F* < 1, *P* = .61, η^2^ = .01) or time (*F*_1,31_ = 2.00, *P* = .17, η^2^ = .06), nor their interaction (*F*_1,31_ = 1.82, *P* = .19, η^2^ = .06). These nonsignificant main and interaction effects were repeated for each of the social problem subscales except for impulsive/carelessness, where a significant time main effect was evident (*F*_1,31_ = 9.72, *P* = .004, η^2^ = .24). Irrespective of participation in the intervention, the young people reported less of this problem-solving behavior at time 2 (mean 7.06, SD 3.43) than they did at time 1 (mean 9.18, SD 4.58). The mixed-model ANOVA on participants’ attitudes toward illness resulted in nonsignificant main effects of time (*F*_1,33_ = 3.23, *P* = .08, η^2^ = .09) and group (*F* < 1, η^2^ = .01), as well as their interaction (*F*_1,33_ = 1.26, *P* = .27, η^2^ = .04).

For the spirometry results, both main effects of group (*F*_1,35_ = 1.69, *P* = .20, η^2^ = .05) and time (*F* < 1, *P* = .65, η^2^ = .01) were nonsignificant, as was their interaction (*F* < 1, *P* = .64, η^2^ = .01).

Despite these nonsignificant findings, we conducted further analyses to explore more fully and specifically the hypotheses of interest. These analyses incorporated examination of group differences on participants’ change scores (ie, difference in scores between time 1 and time 2), differences across time for each of the BEO and wait-list groups separately, and a priori levels of clinically significant (as opposed to statistically significant) change.

The findings from the analyses of change scores are summarized in Table 3. There were no significant group differences for change scores across all outcome measures. However, further analyses investigating the significance of the differences across time for the BEO and wait-list groups separately did identify some noteworthy findings. In line with the stated hypotheses, the depression scores for the BEO group following intervention (mean 9.53, SD 7.54) were significantly lower than reported at baseline (mean 13.67, SD 10.52; *F*_1,33_ = 4.38, *P* = .04, η^2^ = .12). Such was not the case for the wait-list group (time 1: mean 16.41, SD 15.64; time 2: mean 17.09, SD 13.24; *F* < 1, *P* = .54, η^2^ = .01).

For the SPSI-R:SF, follow-up analyses indicated that those in the BEO group were significantly less likely to report an impulsive/careless problem-solving style following their intervention (mean 5.88, SD 2.89) than at time 1 (mean 8.44, SD 4.10; *F*_1,31_ = 6.80, *P* = .01, η^2^ = .18). The wait-list group showed no significant difference in this approach to problem solving across the same time period (time 1: mean 9.88, SD 5.01; time 2: mean 8.18, SD 3.61; *F*_1,31_ = 3.20, *P* = .08, η^2^ = .09).

Further, the participants in the BEO group reported a significantly better attitude toward their illness following the intervention (mean 3.61, SD 0.60) than at baseline (mean 3.29, SD 0.64; *F*_1,33_ = 4.40, *P* = .04, η^2^ = .12). There was no significant difference in the wait-list group (*F* < 1, *P* = .64, η^2^ = .01).

 In some settings, differences between groups may be considered of clinical significance despite nonsignificant statistical differences. A common decision criterion for clinical significance is a change in scores (or difference between the groups) equal to one-half of the average of the pooled standard deviation [20]. Based on this criterion, there was evidence of trends toward clinical significance in group differences for depression, impulsive/careless problem-solving style, and attitudes toward illness with improvements supporting the BEO group.

We analyzed responses to the Intervention Satisfaction Scale questionnaire to gauge the participants’ perceptions of the BEO program following their completion of the online modules. Almost all (18/19, 95% of participants) reported that they were happy to do the program and “thoroughly enjoyed” it (15/19, 79%). Most (18/19, 95%) stated that they would highly recommend the program to others. Only 2 participants dropped out of the program (1 family relocated) and all participants who remained in the project completed all 6 online modules.

**Table 2:** Descriptive statistics on outcome measures for children and adolescents for Breathe Easier Online (BEO) intervention and wait-list control conditions

	Outcome measure		BEO Intervention		Wait-list	
			Mean	SD	Mean	SD
**CES-DC**^a^						
	Time 1		13.67	10.52	16.41	15.64
	Time 2		9.53	7.54	17.09	13.24
**SPSI-R:SF**^b^						
	Positive problem orientation					
		Time 1	10.44	4.44	11.35	3.14
		Time 2	10.28	3.74	10.65	4.66
	Negative problem orientation					
		Time 1	5.88	4.90	5.82	2.88
		Time 2	4.06	3.09	6.85	5.68
	Rational problem solving					
		Time 1	8.06	4.42	9.24	4.91
		Time 2	8.82	5.93	8.88	3.68
	Impulsive/careless style					
		Time 1	8.44	4.10	9.88	5.01
		Time 2	5.88	2.89	8.18	3.61
	Avoidance style					
		Time 1	7.19	4.71	7.06	3.17
		Time 2	6.13	4.43	6.48	5.03
	Overall score					
		Time 1	11.40	3.29	11.56	1.86
		Time 2	12.62	2.65	11.59	2.86
**CATIS**^c^						
	Time 1		3.29	0.64	3.31	0.76
	Time 2		3.61	0.60	3.38	0.72
**FEV1pred**^d^ (%)						
	BEO		83.17	18.88	74.19	26.31
	Wait-list		83.19	17.20	71.98	32.52

^a^ Center for Epidemiologic Studies Depression Scale for Children (higher scores reflect greater depressive symptomatology).

^b^ Social Problem-Solving Inventory–Revised (Short Form) (higher scores reflect greater use of that problem-solving style).

^c^ Child Attitude Toward Illness Scale (higher scores reflect a more positive attitude toward the illness).

^d^ Predicted forced expiratory volume in 1 second.

**Table 3:** Descriptive statistics on change scores (time 1 to time 2 differences) of outcome measures for children and adolescents for Breathe Easier Online (BEO) intervention and wait-list control conditions

Outcome measure		Change scores				*P* value^a^	Effect size
		BEO Intervention		Wait-list			
		Mean	SD	Mean	SD		
CES-DC^b^		–4.14	7.56	0.68	13.29	.19	.05
**SPSI-R:SF**^c^							
	Positive problem orientation	–0.16	4.00	–0.71	5.38	.74	.01
	Negative problem orientation	–1.81	4.20	1.03	4.94	.09	.09
	Rational problem solving	0.81	4.15	–0.41	3.32	.36	.03
	Impulsive/careless style	–2.56	3.16	–1.71	4.54	.54	.01
	Avoidance style	–1.06	5.08	–0.59	5.40	.79	.01
	Overall score	1.22	2.62	0.03	2.45	.19	.06
CATIS^d^		0.32	0.57	0.07	0.73	.27	.04
FEV_1_pred^e^		0.03	13.47	–2.21	15.19	.64	.01

^a^ *P* value comparing group differences on change scores.

^b^ Center for Epidemiologic Studies Depression Scale for Children (negative scores represent fewer symptoms of depression at time 2 than at time 1).

^c^ Social Problem-Solving Inventory (negative scores represent less of that style at time 2 than at time 1).

^d^ Child Attitude Toward Illness Scale (positive change scores represent a more positive attitude at time 2 than at time 1).

^e^ Predicted forced expiratory volume in 1 second.

## Discussion

BEO is an Internet-based intervention designed to improve the well-being of children and adolescents with a chronic respiratory condition. Although there were no significant group differences following the intervention, there was evidence, albeit preliminary and tentative, of the efficacy of the program in improving attitudes toward illness, reducing depression symptoms, and decreasing maladaptive social problem solving (impulsive/careless style) for the participants in the BEO group following their intervention. The program was met with considerable enthusiasm from the participants and their parents. The majority of families reported they would definitely recommend it to others. The attrition rate was very low, as participants enjoyed interacting on the specifically created website.

### Comparisons with Prior Work

The present study extends on previous research [21-24] by demonstrating the initial efficacy and feasibility of Internet-based interventions with young people with chronic health conditions. However, many of the previously reported interventions have not specifically addressed the young person’s psychosocial well-being but have focused on symptom monitoring [21], pain treatment [25], health care [26], and education [23]. In fact, where psychosocial well-being was an outcome, the findings have been equivocal [21,22]. Although acknowledging the methodological, intervention, and illness differences that exist between the present study and this past research, it is important to note that our study is unique in that we found modest improvements in problem solving and attitudes toward illness and lowering depression following our online intervention.

### Limitations

Despite the promising findings, our study has some limitations that must be recognized. The nonsignificant group differences across all outcome measures need to be interpreted in light of several factors. It may be that the intervention, itself, was not sufficiently focused or presented on a platform that would engage the participants in a way that would lead to hypothesized improvements. While necessarily directed at set and established problems, the online modules might have provided more room for participant-own identification of their problems followed by therapist-guided group identification of resolutions. This would have created a greater relevance, engagement, and interaction with the modules. The sample size was small, and this may have affected our ability to detect true group differences across other outcome measures (ie, statistical power). Although statistically nonsignificant, some of our findings were encouraging, indicating trends that a larger sample size might lead to significant results. Another limitation concerned the participants’ baseline well-being scores. These were not at clinical levels (except for the depression scores in the wait-list group), and this may have hindered the likelihood of improvements showing following the intervention. There is some evidence that interventions for participants not within a clinical range do not produce changes of significance in symptoms [27]. Finally, the children progressed unevenly and at various speeds through the online modules with life events (eg, holidays) disrupting progress. This is an acknowledged problem with Internet interventions with children [28] and can have an influence on their impact.

### Future Research

This study has provided pilot data for future work, as the small sample size renders this study underpowered. Also, the findings of the current pilot study highlight several avenues for future research. Despite the participants’ engagement with the Web-based modules, anecdotal feedback suggests that they were too text intensive. Future research may investigate the efficacy of Internet-based interventions using different modalities (text, picture, or video) across different age groups to find the optimal age-related combination. While the children and adolescents in our BEO group demonstrated positive gains, the question as to whether these gains compare favorably with those of face-to-face interventions remains unanswered, as does the question of the maintenance of any gains in well-being. Future research that makes direct comparisons with a face-to-face intervention group is necessary to ensure that the online environment does overcome some of the face-to-face intervention barriers (eg, accessibility) but not at the expense of gains in well-being.

### Conclusions

The Internet offers an exciting possibility for intervention with children and adolescents with chronic respiratory conditions, as it can be interactive, engaging, and fun and can provide unique opportunities for online peer and professional support that can continue once the intervention has been completed. Moreover, it can overcome some of the barriers and impediments found in face-to-face interventions. Our findings are promising but also highlight the need for further research attention toward specifically designed online programs as an intervention with children and adolescents with chronic health conditions.

## Multimedia Appendix 1

CONSORT-EHEALTH (V1.6) checklist [29].

## Abbreviations

ANOVA: analysis of variance
BEO: Breathe Easier Online
CATIS: Child Attitude Toward Illness Scale
CES-DC: Center for Epidemiologic Studies Depression Scale for Children
FEV1: forced expiratory volume in 1 second
PACE: problem, alternative solutions, consequences, execute
SPSI-R:SF: Social Problem-Solving Inventory–Revised (Short Form)
