# Supplementary material for: Breathe Easier Online: Evaluation of a Randomized Controlled Pilot Trial of an Internet-Based Intervention to Improve Well-being in Children and Adolescents With a Chronic Respiratory Condition
Source: J Med Internet Res. 2012 Feb 8;14(1):e23. doi: 10.2196/jmir.1997 (PMC3374545; doi:10.2196/jmir.1997)
Supplement: Supplementary file 1 [file jmir_v14i1e23_app1.pdf]

CONSORT-EHEALTH Checklist V1.6 Report

Manuscript Number 1997
Date completed 8/25/2011 14:50:29
by Peter Newcombe

"Breathe Easier Online: An evaluation of a randomised controlled pilot trial of an Internet-based intervention to improve well-being in children and adolescents with a chronic respiratory condition"

TITLE

1a-i) Identify the mode of delivery in the title
" Breathe Easier Online: An evaluation of a randomised controlled pilot trial of an Internet-based intervention to improve well-being in children and adolescents with a chronic respiratory condition"

1a-ii) Non-web-based components or important co-interventions in title
There are no important non-web-based components or co-interventions in the research

1a-iii) Primary condition or target group in the title
"in children and adolescents with a chronic respiratory condition"

ABSTRACT

1b-i) Key features/functionalities/components of the intervention and comparator in the METHODS section of the ABSTRACT
"This randomised controlled pilot trial was conducted to evaluate the efficacy of Breathe Easier Online (BEO), an Internet-based problem solving program with minimal facilitator involvement to improve psychosocial well-being in children and adolescents with a chronic respiratory condition." p. 2
"Forty-two socially isolated children and adolescents (18 males), aged between 10 and 17 years were randomly assigned to either a BEO (final n = 19) or a wait-list (WL, final n = 20) condition." p. 2

1b-ii) Level of human involvement in the METHODS section of the ABSTRACT
"an Internet-based problem solving program with minimal facilitator involvement" p. 2

1b-iii) Open vs. closed, web-based (self-assessment) vs. face-to-face assessments in the METHODS section of the ABSTRACT
"Paper-and-pencil questionnaires were completed at the hospital when participants attended a briefing session at baseline (Time 1) and in their homes after the intervention for the BEO group or a matched 9-week time period for the WL group (Time 2). " p. 2

1b-iv) RESULTS section in abstract must contain use data
"Forty-two socially isolated children and adolescents (18 males), aged between 10 and 17 years were randomly assigned to either a BEO (final n = 19) or a wait-list (WL, final n = 20) condition. Three participants (two from BEO and one from WL) did not complete the intervention. " p. 2
"For the primary outcome measures, there were no significant Group differences on depression, P = .167, or social problem solving, P = .931. However, those in the BEO group reported significantly lower depression, P = .044, less impulsive/careless problem solving, P = .044, and an improvement in positive attitude towards their illness, P = .014, following the online intervention. " p. 2

1b-v) CONCLUSIONS/DISCUSSION in abstract for negative trials
"Although there were no significant group differences on primary outcome measures, our pilot data provides tentative support for the feasibility (acceptability and user satisfaction) and initial efficacy of an Internet-based intervention for improving well-being in children and adolescents with a chronic respiratory condition." p. 3

INTRODUCTION

2a-i) Problem and the type of system/solution
"Children and adolescents with a chronic respiratory condition often feel different to their healthy peers due to the necessity of a daily treatment regime; can have trouble maintaining friendships because of school absences[4]; and can experience psychological difficulties such as anxiety, depression, and poor quality of life (for review, see [5]). They also tend to be involved in risky behaviors such as smoking and make poor lifestyle choices." P. 5
"While face-to-face psychological interventions for adolescents with chronic health conditions are available in most hospitals, the limited availability and accessibility of such programs is a substantial barrier to more widespread uptake of routine psychosocial treatment or preventative interventions. The Internet offers a dynamic, interactive medium for providing information, changing attitudes and behaviour, and enhancing social support[12]. " p. 6

2a-ii) Scientific background, rationale: What is known about the (type of) system
"Based on the finding that multicomponent interventions are more successful than educational interventions alone[9], our study incorporated social support and problem-solving skills to improve psychosocial well-being in children and adolescents with a chronic respiratory condition. The problem-solving paradigm was based on D'Zurilla and Nezu (1999) who framed problem solving as a cognitive-behavioural process whereby individuals endeavour to focus their coping efforts on altering the problematic nature of the situation, their reactions to the situation, or both[15]. An Internet intervention, Breathe Easier Online (BEO), was developed specifically to improve social support and problem solving skills. " p. 7

METHODS

3a) CONSORT
"We hypothesized that, in this pilot RCT, those receiving the BEO intervention would show improved psychological well-being (as evidenced by lower depression scores) and problem-solving skills following intervention compared to those in a wait-list control group. We also anticipated positive secondary health-related outcomes for the BEO group including an improved attitude towards their illness and improved treatment adherence." pp. 7-8

3b-i) Bug fixes, Downtimes, Content Changes
There were no major bug fixes or changes in functionality or content made throughout the course of the trial

4a-i) Computer / Internet literacy
"Exclusion criteria were children who were unable to use a computer, those with an underlying psychiatric disorder or recent (<3 months) hospitalization." p. 8

4a-ii) Open vs. closed, web-based vs. face-to-face assessments:
"Paper-and-pencil questionnaire packages were completed at the hospital or at participants' homes at two time points (Time 1: baseline/pre-intervention and Time 2: post-intervention) for the BEO condition and at time equivalents to pre- and post-intervention (9 weeks later) for the WL group." p. 9

Participants were known to the research team as they were recruited through the hospital and allocated with a laptop computer as part of the trial. Research team were in email/phone contact with participants to check on progress through the modules, for following up on completion of questionnaires and to address any computer hardware issues.

4a-iii) Information giving during recruitment
Parents of eligible children were advised of the trial by hospital staff when they attended the hospital. They were provided with a written information sheet and an informed consent sheet to be completed if they wished to participate.

"Potential participants and their families were identified through hospital records by a research nurse at the hospital between July 2008 and November 2009, and invited to participate. " p. 8

4b-i) Report if outcomes were (self-)assessed through online questionnaires
"Paper-and-pencil questionnaire packages were completed at the hospital or at participants' homes at two time points (Time 1: baseline/pre-intervention and Time 2: post-intervention) for the BEO condition and at time equivalents to pre- and post-intervention (9 weeks later) for the WL group." p. 9
"Intervention satisfaction scale. This was a purpose-created self-report scale designed to explore the participants' opinions and satisfaction with the intervention as well as seeking feedback at Time 2." p. 10

4b-ii) Report how institutional affiliations are displayed
All institutions affiliated with the trial are listed on the information sheet and consent form and shown at the bottom of the screen on the Internet-based program. See p. 12 for Screen Shot of a web-page.

5-i) Mention names, credential, affiliations of the developers, sponsors, and owners
Sponsor/grant provided is listed in the acknowledgement section of the ms and research team with affiliations are listed on the title page of the ms

5-ii) Describe the history/development process
The program was developed for evaluation in this pilot trial. The research team provided input into the development of the content and a web designer from the University of Queensland provided input into the design elements.

5-iii) Revisions and updating
Given the pilot nature of this trial, no revisions or updating have been undertaken to date.

5-iv) Quality assurance methods
Hospital staff reviewed all health-based material and psychology researchers reviewed all content of the intervention for accuracy and quality of information provided.

5-v) Ensure replicability by publishing the source code, and/or providing screenshots/screen-capture video, and/or providing flowcharts of the algorithms used
Screen shot of the program is shown on p. 12.
5-vi) Digital preservation
The program is archived on password-protected server within the School of Psychology, The University of Queensland
5-vii) Access
Participants accessed the program from their homes using their unique password and the lap top computer provided to them. They did not have to pay, and were not paid, to use the program

"My talk. This component of the website provided opportunities for BEO participants to communicate with each other. This communication could be either asynchronous (discussion board, email) or synchronous (Instant Messenger)." p. 11
5-viii) Mode of delivery, features/functionalities/components of the intervention and comparator, and the theoretical framework
"Our study incorporated social support and problem-solving skills to improve psychosocial well-being in children and adolescents with a chronic respiratory condition. The problem-solving paradigm was based on D'Zurilla and Nezu (1999) who framed problem solving as a cognitive-behavioural process whereby individuals endeavour to focus their coping efforts on altering the problematic nature of the situation, their reactions to the situation, or both[15]. " p. 7
"The intervention consisted of two parts – structured modules that the participants completed on a weekly basis to improve their social problem solving skills and an online community that incorporated both synchronous and asynchronous communication opportunities." p. 7
"My talk. This component of the website provided opportunities for BEO participants to communicate with each other. This communication could be either asynchronous (discussion board, email) or synchronous (Instant Messenger)." p. 11
5-ix) Describe use parameters
Participants were allowed self-paced access to the program, although it was suggested that the complete the modules and homework on a weekly basis. They could review previous sessions and use the communication facilities as often as they wanted. They were requested to keep their diary on a daily basis.

"My work. This section of the website contained the six modules that formed the focal intervention. The modules were based on D'Zurilla's problem-solving theory[15] and provided interactive online skills training that targeted problems in general in addition to illness-specific problems. The online package followed a "PACE" principle for solving problems: Problem identification, Alternative solution generation, Consequences of each alternative solution, Execute solution and evaluate.
Daily diary. This section contained a checklist where participants noted the medications they had taken each day. They also recorded how often they conversed with other participants in the program. " p. 11
5-x) Clarify the level of human involvement
The intervention was accessed through the Internet. Participants had email or phone contact with research staff if they experienced any problems with the operation of the computer or the modem. Research staff also had contact with the participants when monitoring progress through the modules, moderating the communication tools, and administering the self-report questionnaires.
5-xi) Report any prompts/reminders used
Progress through modules was monitored weekly by the research team with email or phone prompts/reminders used as needed.
5-xii) Describe any co-interventions (incl. training/support)
There were no co-interventions provided in addition to the targeted intervention. Participants however, continued treatment as usual for their respiratory conditions.
6a-i) Online questionnaires: describe if they were validated for online use and apply CHERRIES items to describe how the questionnaires were designed/deployed
Online questionnaires were not used in the current trial.
6a-ii) Describe whether and how "use" (including intensity of use/dosage) was defined/measured/monitored
All participants in the study who completed the program completed all modules.
Completion of the modules was the primary focus of this study.
6a-iii) Describe whether, how, and when qualitative feedback from participants was obtained
"Intervention satisfaction scale. This was a purpose-created self-report scale designed to explore the participants' opinions and satisfaction with the intervention as well as seeking feedback at Time 2. Eight items (e.g., "Would you recommend the program to others?") were rated on a four-point Likert-scale (1='yes, very much so', 2='yes, for the most part', 3='no, not really', 4='no, not at all'). A final open-ended item allowed for any general comments or suggestions about the BEO intervention." p. 10
7a-i) Describe whether and how expected attrition was taken into account when calculating the sample size
"Sample size was based on an expected medium effect size of change in psychosocial measures for the intervention group with statistical power set at 0.90 and α=0.05." p. 13

"An intention-to-treat approach to analyses was adopted with all participants included in the final analysis. The intention-to-treat analysis (missing values replaced with linear trend) ensured that those participants who completed Time 1 measures and the intervention (but did not complete all Time 2 measures) were included in the analyses." p. 13
7b) CONSORT
No interim analyses were completed and there were no stopping guidelines.
8a) CONSORT
"Order of random allocation was pre-determined via a computer program and was unknown to and concealed from the research staff." p. 8
8b) CONSORT
There were no restrictions to randomisation. Once participants were recruited to the study they were randomised to condition.
9) CONSORT
"Order of random allocation was pre-determined via a computer program and was unknown to and concealed from the research staff." p. 8
10) CONSORT
Hospital staff who were not part of the research project generated the random allocation sequence.
11a-i) Specify who was blinded, and who wasn't
Research staff were aware of which condition participants were in. Participants were aware of their condition status.
11a-ii) Discuss e.g., whether participants knew which intervention was the "intervention of interest" and which one was the "comparator"
Participants were aware whether the condition they were in was the intervention of interest or the comparator.
11b) CONSORT
Not relevant to this trial
12a) CONSORT
" Linear mixed models were used to assess the effects of the intervention on outcomes using a mixed-model ANOVA approach with Time (Time 1 vs. Time 2) as a within-subjects factor and Group (BEO vs. WL) as a between-subjects factor. Follow-up analyses were conducted in line with the stated hypotheses. The criterion for statistical significance was set a two-tailed value of ≤ 0.05." p. 13
12a-i) Imputation techniques to deal with attrition / missing values
As there were only 3 participants who did not complete the study (2 from intervention and 1 from control, intention-to-treat was judged to be an appropriate approach).

"An intention-to-treat approach to analyses was adopted with all participants included in the final analysis. The intention-to-treat analysis (missing values replaced with linear trend) ensured that those participants who completed Time 1 measures and the intervention (but did not complete all Time 2 measures) were included in the analyses." p. 13
12b) CONSORT
"Despite these non-significant findings, further analyses were conducted to explore more fully and specifically the hypotheses of interest. These analyses incorporated examination of Group differences on participants' change scores (i.e., difference scores between Time 1 and Time 2), differences across Time for each of the BEO and WL groups separately, and apriori levels of clinically significant (as opposed to statistically significant) change." p. 18

RESULTS

13a) CONSORT

See flow chart in Figure 1, p. 15.

13b) CONSORT

See flow chart in Figure 1, p. 15.

13b-i) Attrition diagram

Given there were only three participants who did not complete the trial, an attrition diagram was not considered necessary.

14a) CONSORT

" Potential participants and their families were identified through hospital records by a research nurse at the hospital between July 2008 and November 2009, and invited to participate." p. 8

"Paper-and-pencil questionnaire packages were completed at the hospital or at participants' homes at two time points (Time 1: baseline/pre-intervention and Time 2: post-intervention) for the BEO condition and at time equivalents to pre- and post-intervention (9 weeks later) for the WL group. At Time 2, the WL group was invited to participate in the intervention." p. 9

14a-i) Indicate if critical "secular events" fell into the study period

There were no significant secular events during the curse of the study period of the trial.

14b) CONSORT

The trial was not ended or stopped early.

15) CONSORT

Demographic characteristics for each group are shown in Table 1, p. 16

15-i) Report demographics associated with digital divide issues

See Table 1, p. 16

16-i) Report multiple "denominators" and provide definitions

See flow chart in Figure 1, p. 15.

16-ii) Primary analysis should be intent-to-treat

"An intention-to-treat approach to analyses was adopted with all participants included in the final analysis. The intention-to-treat analysis (missing values replaced with linear trend) ensured that those participants who completed Time 1 measures and the intervention (but did not complete all Time 2 measures) were included in the analyses." p. 13

17a) CONSORT

"The descriptive data for the mixed model ANOVA analyses are presented in Table 2 showing primary outcomes of depressive symptoms and social problem solving skills as well as secondary outcomes of attitude towards illness, and spirometry (as an indicator of treatment adherence). The results for the analyses of depression scores revealed neither significant Group, $F(1,33) = 2.00$, $P = .167$, $\eta^2 = .06$, or Time, $F < 1$, $P = .347$, $\eta^2 = .03$, main effects nor a significant interaction, $F(1,33) = 1.76$, $P = .193$, $\eta^2 = .05$. Similar results were found for full scale scores on social problem with neither significant main effects for Group, $F < 1$, $P = .610$, $\eta^2 = .01$, or Time, $F(1,31) = 2.00$, $P = .167$, $\eta^2 = .06$, nor their interaction, $F(1,31) = 1.82$, $P = .187$, $\eta^2 = .06$. These non-significant main and interaction effects were repeated for each of the social problem subscales except for Impulsive / Carelessness where a significant Time main effect was evident, $F(1,31) = 9.72$, $P = .004$, $\eta^2 = .24$. Irrespective of participation in the intervention, the young people reported less of this problem solving behavior at Time 2 ($M = 7.06$, $SD = 3.43$) than they did at Time 1 ($M = 9.18$, $SD = 4.58$). The mixed model ANOVA on children's attitudes towards illness resulted in non-significant main effects of Time, $F(1,33) = 3.23$, $P = .081$, $\eta^2 = .09$, or Group, $F < 1$, $\eta^2 = .01$, as well as their interaction, $F(1,33) = 1.26$, $P = .269$, $\eta^2 = .04$." p. 14

17a-i) Presentation of process outcomes such as metrics of use and intensity of use

The key aspect of the trial was completion of the modules which was monitored by the research team. usage and intensity was not focal to the research and therefore not reported.

17b) CONSORT

There we no binary outcomes assessed in the current trial.

18) CONSORT

"Despite these non-significant findings, further analyses were conducted to explore more fully and specifically the hypotheses of interest. These analyses incorporated examination of Group differences on participants' change scores (i.e., difference scores between Time 1 and Time 2), differences across Time for each of the BEO and WL groups separately, and apriori levels of clinically significant (as opposed to statistically significant) change. The findings from the analyses of change scores are summarized in Table 3. As can be seen from this table, there were no significant Group differences for change scores across all outcome measures." p. 18

18-i) Subgroup analysis of comparing only users

"However, further analyses investigating the significance of the differences across Time for the BEO and WL groups separately did identify some noteworthy findings. In line with the stated hypotheses, the depression scores for the BEO group following intervention ($M = 9.53$, $SD = 7.55$) were significantly lower than reported at baseline ($M = 13.67$, $SD = 10.52$), $F(1,33) = 4.38$, $P = .044$, $\eta^2 = .12$. Such was not the case for the WL group (Time 1: $M = 16.42$, $SD = 15.64$; Time 2: $M = 17.09$, $SD = 13.24$), $F < 1$, $P = .544$, $\eta^2 = .01$.

For the Social Problem Solving Inventory, follow-up analyses indicated that those in the BEO group were significantly less likely to report an impulsive/careless problem solving style following their intervention ($M = 5.88$, $SD = 2.89$) than at Time 1 ($M = 8.44$, $SD = 4.10$), $F(1,31) = 6.80$, $P = .014$, $\eta^2 = .18$. The WL group showed no significant difference in this approach to problem solving across the same time period (Time 1: $M = 9.88$, $SD = 5.01$; Time 2: $M = 8.18$, $SD = 3.61$), $F(1,31) = 3.20$, $P = .083$, $\eta^2 = .09$.

Further, the children in the BEO group reported a significantly better attitude towards their illness following the intervention ($M = 3.61$, $SD = 0.60$) than at baseline ($M = 3.29$, $SD = 0.64$), $F(1,33) = 4.40$, $P = .044$, $\eta^2 = .12$. There was no significant difference in the WL group, $F < 1$, $P = .642$, $\eta^2 = .01$.
" p 18.

19) CONSORT

There were no important harms or unintended effects in either intervention or control group during the trial.

19-i) Include privacy breaches, technical problems

There were no privacy breaches or major technical problems during the trial.

19-ii) Include qualitative feedback from participants or observations from staff/researchers

"Responses to the Intervention Satisfaction Questionnaire were analysed to gauge the participants' perceptions of the BEO program following their completion of the online modules. Almost all (95% of participants) reported that they were happy to do the program and "thoroughly enjoyed" it (83%). Most (90%) stated that they would highly recommend the program to others. Only two participants dropped out of the program (one family relocated) and all participants who remained in the project completed all six online modules." p. 20

DISCUSSION

20-i) Typical limitations in ehealth trials

"It may be that the intervention, itself, was not sufficiently focused or presented on a platform that would engage the participants in a way that would lead to hypothesized improvements. Whilst necessarily directed at set and established problems, the online modules might have provided more room for participant-own identification of their problems followed by therapist-guided group identification of resolutions. This would have created a greater relevance, engagement, and interaction with the modules. The sample size was small and this may have affected our ability to detect true group differences across other outcome measures (i.e., statistical power). Although statistically non-significant, some of findings were encouraging indicating trends that a larger sample size might lead to significant results. Another limitation concerned the participants' baseline well-being scores. These were not at clinical levels (except for the depression scores in the WL group) and this may have hindered likelihood of improvements showing following the intervention. There is some evidence that interventions for participants not within a clinical range do not produce changes of significance in symptomology[27]. Finally, the children progressed unevenly and at various speeds through the online modules with life events (e.g., holidays) disrupting progress. This is an acknowledged problem with Internet interventions with children[28] and can have an influence on their impact." pp. 21-22

21-i) Generalizability to other populations

"Our findings are promising but also highlight the need for further research attention towards specifically designed online programs as an intervention with children and adolescents with chronic health conditions." p. 23

21-ii) Discuss if there were elements in the RCT that would be different in a routine application setting

“Whilst the children in our BEO group demonstrated positive gains, the question as to whether these gains compare favourably with face-to-face interventions remains unanswered as does the question of the maintenance of any gains in well-being. Future research that makes direct comparisons with a face-to-face intervention group is necessary to ensure that the online environment does overcome some of the face-to-face intervention barriers (e.g., accessibility) but not at the expense of gains in well-being.” p. 22

22-i) Restate study questions and summarize the answers suggested by the data, starting with primary outcomes and process outcomes (use)

“Although there were no significant Group differences following the intervention, there was evidence, albeit preliminary and tentative, of the efficacy of the program in improving attitudes towards illness, reducing depression symptoms, and decreasing maladaptive social problem solving (impulsive/careless style) for the participants in the BEO group following their intervention.” p. 20

22-ii) Highlight unanswered new questions, suggest future research

“This study has provided pilot data for future work as the small sample size renders this study under-powered. Also, the findings of the current pilot study highlight a number of avenues for future research. Despite engagement with the web-based modules, anecdotal feedback suggests that they were too text-intensive. Future research may investigate the efficacy of Internet-based interventions utilizing different modalities (text, picture, video) across different age groups to find the optimal age-related combination. Whilst the children in our BEO group demonstrated positive gains, the question as to whether these gains compare favourably with face-to-face interventions remains unanswered as does the question of the maintenance of any gains in well-being. Future research that makes direct comparisons with a face-to-face intervention group is necessary to ensure that the online environment does overcome some of the face-to-face intervention barriers (e.g., accessibility) but not at the expense of gains in well-being.” p. 22

Other information

23) CONSORT

“Trial registration: Australian New Zealand Clinical Trials Registry Number: ACTRN12610000214033; http://www.anzctr.org.au/trial_view.aspx?ID=308074 (Archived by WebCite at URL http://www.webcitation.org/63BL55mXH).” p. 3

24) CONSORT

“Trial registration: Australian New Zealand Clinical Trials Registry Number: ACTRN12610000214033; http://www.anzctr.org.au/trial_view.aspx?ID=308074 (Archived by WebCite at URL http://www.webcitation.org/63BL55mXH).” p. 3

25) CONSORT

"Telstra Foundation and Royal Children's Hospital Foundation (Grant Number 10237)" p. 24

X26-i) Comment on ethics committee approval

“Ethical approval was granted by the Behavioral and Social Sciences Ethical Review Committee at the University of Queensland and the Human Ethics Committee of the Royal Children’s Hospital, Brisbane.” p. 8

x26-ii) Outline informed consent procedures

“ Informed consent was obtained from both the parent and participant in those aged ≥12 years and from parents alone in children aged <12 years.” p. 8

X26-iii) Safety and security procedures

Access to the program was password protected to protect participants’ privacy. Research staff were available by email or phone to deal with any issues that arose for participants. Assessment data collected from participants was stored confidentially.

X27-i) State the relation of the study team towards the system being evaluated

There are no financial conflicts of interest in the trial. The BEO program evaluated in the trial was developed by the authors.
